# Supplementary material for: Collaborative intelligence in AI: Evaluating the performance of a council of AIs on the USMLE
Source: PLOS Digit Health. 2025 Oct 9;4(10):e0000787. doi: 10.1371/journal.pdig.0000787 (PMC12510544; doi:10.1371/journal.pdig.0000787)
Supplement: S2 File — (ZIP) [file pdig.0000787.s002.zip › S2 Data Raw data files containing unprocessed question inputs and LLM outputs including transcripts of deliberations/STEP3_Question-86-1_Type-MC-NJ.docx]

**Facilitator** **[Round 1]:** A 45-year-old man returns to the office for ongoing treatment of tuberculous pericarditis. Pericardiocentesis 9 weeks ago showed a serosanguineous effusion; culture of the effusion was positive for Mycobacterium tuberculosis, and therapy with isoniazid, rifampin, and ethambutol was initiated. During the past 2 weeks, he has had increasing shortness of breath on minimal exertion, cough, pain in the right upper quadrant, and swelling of the lower extremities. BMI is 26 kg/m² . Vital signs today are temperature 37.0°C (98.6°F), pulse 100/min, respirations 22/min, and blood pressure 105/65 mm Hg. Physical examination discloses 10 cm of jugular venous distention. Lungs are clear to auscultation and percussion. Cardiac examination discloses distant but normal heart sounds. The liver is tender and is palpated 3 cm below the right costal margin. There is 2+ to 3+ pitting edema from the knees distally. HIV antibody test is negative. ECG shows nonspecific ST-T changes. Chest x-ray today is unchanged from the x-ray obtained at the time of diagnosis, which showed resolution of apical and perihilar infiltrates and a normal-sized heart. Which of the following is the most appropriate next step in management? (A) Add furosemide therapy and reevaluate in 4 weeks (B) Add prednisone therapy (C) Add streptomycin and cycloserine therapy (D) Order bronchoscopy for brushings and cytology (E) Order echocardiography

**AI_D:** RESTATING THE QUESTION: The patient, a 45-year-old man, who returned for a routine follow-up visit for his diagnosed condition of tuberculous pericarditis, is presenting with worsening symptoms such as increasing shortness of breath, cough, right upper quadrant pain, and swelling of the lower extremities. The patient is undergoing treatment with isoniazid, rifampin, and ethambutol since his diagnosis 9 weeks ago. The patient has no chest abnormalities on the x-ray, yet there is palpable liver enlargement and lower extremity edema along with increased jugular venous distension. We are sought to determine the ideal course of management. PLANNING: The assumptions in our prognosis are that the treatment plan for tuberculous pericarditis of isoniazid, rifampin, and ethambutol has been adequate. An alternative assumption comes from clinical presentation suggesting ongoing inflammation of the pericardium (shortness of breath, distended jugular veins, distant heart sounds, liver enlargement) and signs of right-side heart failure (edema) despite treatment. If we evaluate the assumption, it might indicate that the treatment has failed to control the inflammation, hence the condition may have worsened viz. constrictive pericarditis. CLINICAL REASONING: Given the patient's presentation, understanding the pathophysiology of tuberculosis pericarditis is critical. Initially, it presents as a pericardial effusion which can then note a progressive thickening and calcification of the pericardium over time leading to constrictive pericarditis. This condition is a result of chronic inflammation that turns the normally pliable pericardium into a rigid shell. This restricts diastolic filling of the heart and, with time, can cause signs of heart failure, as seen in this patient (i.e., edema, hepatic congestion). CONCLUDING: With the new symptoms presented by the patient, detailed evaluation of the cardiac structures with a suitable diagnostic procedure to visualize this constriction is clinically essential. RESPONSE: The correct choice would be to order echocardiography (E), which is the investigation of choice for evaluating the pericardium and confirming the presence of constrictive pericarditis.

**AI_E:** RESTATING THE QUESTION: The patient is a 45 year old male who is currently receiving treatment for Tuberculous Pericarditis; a condition where the pericardium is inflamed due to a Mycobacterium tuberculosis infection. Despite being on treatment (Isoniazid, Rifampin, and Ethambutol), he has been experiencing increasing shortness of breath, cough, pain in the right upper quadrant, and swelling of the lower extremities for the past 2 weeks. He also exhibits signs of fluid overload such as jugular venous distention and edema, and a slightly elevated pulse, suggesting possible heart trouble. Current imaging and lab tests do not show deterioration in the lung health or links to other diseases. We are asked to identify what would be the best course of action in this scenario from the presented options. PLANNING: Looking at the symptoms presented, his findings all point to a form of constrictive pericarditis, which may be a complication of his ongoing tuberculous pericarditis. This disorder restricts the heart's function and could provide a reason for his symptoms. Possible causes of this could be scarring resulting from the inflammation, or it could be a response to the medication he is taking. The potential options presented all address different complications or treatment alterations. CLINICAL REASONING: The patient's situation very much indicates a restrictive pericardial disease. The distended jugular veins, liver congestion, and peripheral edema suggest an issue related to the heart's inability to properly fill during diastole, characteristic of constrictive pericarditis. The history of tuberculous pericarditis makes this a highly plausible possibility as, despite successful antibiotic treatment of the infection, this could leave the pericardium fibrotic, and the scarring could prevent the heart from properly filling with blood; hence the signs of systemic venous congestion. His stable lung status points away from the pericarditis reactivating or progressing despite ongoing therapy. CONCLUDING: The best course of action, in this case, would be to confirm the suspicion of constrictive pericarditis using an echocardiogram. While adjustment to the medication regimen might help manage symptoms, it won't target the current issue being experienced by the patient. Likewise, the use of a bronchoscopy doesn't line up with the patient's clinical picture as his lungs appear to be performing as expected and there's no requirement to test for other possible infections. RESPONSE: Hence, the most appropriate next step in management in this case would be to Order an Echocardiogram (E). A direct imaging test of the heart would help to confirm the diagnosis of Constrictive Pericarditis, and inform further management plans.

**AI_A:** RESTATING THE QUESTION: The question is asking about a 45-year-old male patient who has been diagnosed with tuberculous pericarditis. This is an inflammation of the heart's outer lining caused by Mycobacterium tuberculosis. The patient is currently undergoing treatment with isoniazid, rifampin, and ethambutol yet he still exhibits symptoms such as shortness of breath, cough, pain in right upper quadrant, and edema in the lower extremities. The clinical findings stratify him into a complex case. The management strategies listed cater different approaches and the 'appropriate next step in management' needs to be identified. PLANNING: The management strategies listed contain options to adjust the medication (through adding furosemide, prednisone, or a combination of streptomycin and cycloserine), or assessing the patient's case further (through bronchoscopy or echocardiography). The primary assumption here is that the patient's symptoms, which have increased during the last two weeks, may be directly linked to worsening pericarditis despite therapy, making it necessary to alter the treatment plan or review the case. However, an alternate assumption could be that the patient's recent symptoms are incidental and not a direct indicator of exacerbation of pericarditis. CLINICAL REASONING: The exacerbation of the patient's symptoms presents many possibilities. One could be a worsening of the pericarditis; another could be a complete new pathophysiological process. Given his symptoms of jugular venous distention, distended but normal heart sounds, tender liver, and lower extremity edema, these manifestations are suggestive of right-sided heart failure and referred as constrictive pericarditis. This is a condition where the pericardium becomes thickened and calcified, limiting the heart's ability to expand and fill, which can lead to systemic congestion, and the finding of a distant heart sound reflects the thickening of the pericardium. Moreover, despite anti-tuberculosis treatment, it's unusual for his symptoms to persist unless the disease has progressed due to potential cardiac tamponade or constriction. CONCLUDING: The presence of the aforementioned symptoms, and their potential link to the persistent pericarditis, suggests the necessity of further diagnostic testing to confirm or explore other potential causes. RESPONSE: Therefore, the most appropriate next step in management is option (E) Order echocardiography. The echocardiography is the most definite method to diagnose constrictive pericarditis and to delineate this complication from other possibilities, and would indeed be warranted given the clinical presentation of the patient.

**AI_C:** RESTATING THE QUESTION: The patient, a middle-aged man, came in for a follow-up treatment for a diagnosed condition, tuberculous pericarditis. The condition was confirmed 9 weeks ago when effusion analysis revealed the causal agent: Mycobacterium tuberculosis. He was treated with isoniazid, rifampin, and ethambutol. In the recent couple of weeks, the patient has been experiencing worsening symptoms, including shortness of breath, cough, right upper quadrant pain, and oedema in the lower limbs. Upon physical examination, it was found that he had jugular venous distention, distant but normal heart sounds and tender hepatomegaly. The patient's ECG shows nonspecific ST-T changes, and chest X-ray does not indicate any changes since the time of diagnosis. The patient tested negative to HIV. Subsequently, the need arises to determine the best approach to manage his current condition. PLANNING: Due to the patient's increased symptoms despite therapy, we must evaluate if the current treatment regimen is effective or there is a complication from tuberculous pericarditis, a condition characterized by an inflammation of the pericardium, the fibroelastic sac covering the heart, caused by M. tuberculosis. An assumption can be made that there's a probable complication known as constrictive pericarditis, manifested through the raised jugular venous pressure, hepatomegaly, edema and possibly the nonspecific ST-T changes in the ECG. CLINICAL REASONING: Tuberculous pericarditis is a dire condition, which untreated, can proceed to constrictive pericarditis, with a clinical presentation of jugular venous distention, hepatomegaly and lower limb edema due to decreased cardiac output causing increased venous pressure and fluid retention. Considering the unresponsiveness to the current treatment and the appearance of such symptoms, constrictive pericarditis appears a likely diagnosis. For an accurate diagnosis and effective treatment plan, imaging studies of the heart need to be performed. CONCLUDING: Given the above clinical reasoning, it is crucial to establish the presence of constrictive pericarditis, which has likely developed due to unresponsive tuberculous pericarditis. This would help plan a more aggressive treatment plan to improve the patient's condition. RESPONSE: From the options provided, the most appropriate course of action would be to order an echocardiography (E). This diagnostic tool allows for visual imaging of the heart, providing an accurate and efficient means to establish the development of constrictive pericarditis. Other options are not directly addressing the development of constrictive pericarditis and might not provide the needed improvements in patient's condition.

**AI_B:** (1) RESTATING THE QUESTION: This clinical vignette describes a 45 year old male who is being treated for tuberculous pericarditis. He had a serosanguineous pericardial effusion nine weeks ago that was positive for mycobacterium tuberculosis. After therapy with isoniazid, rifampin, and ethambutol was initiated, he returned to the doctor's office with new complaints of increasing shortness of breath on minimal exertion, cough, right upper quadrant pain, and lower extremity edema. His vital signs and physical examination findings such as jugular venous distension, enlarged tender liver, and pitting edema suggest systemic disruption likely due to his tuberculous pericarditis. His ECG shows nonspecific ST-T changes and a chest x-ray is found unchanged from the previous time indicating normal-sized heart and resolved pulmonary infiltrates. The question asks for the optimal management strategy for his current condition given the choice of (A) additional furosemide therapy, (B) addition of prednisone, (C) addition of streptomycin and cycloserine, (D) performance of a bronchoscopy for brushings and cytology, and (E) an echocardiography. (2) PLANNING: Let's identify the assumptions. First, we carry the assumption that his new set of symptoms are related to his tuberculous pericarditis. Second, there's the assumption that these new symptoms suggest a change in the disease state or an unsuccessful treatment. For each of these assumptions, it's crucial to generate alternatives. For the first one, the alternate assumption could be that these new symptoms are indicative of an entirely different pathology independent of his pericardial tuberculosis. For the second assumption, an alternate assumption could be that these symptoms represent an immune response to the bacterial infection rather than a direct effect of the bacterium. (3) CLINICAL REASONING: Considering the patient's symptoms and physical examination findings, the picture is leaning more towards cardiac tamponade. The elevated jugular venous pressure, distant heart sounds, tender liver enlargement (hepatic congestion) and dependent edema are traditionally part of Beck's triad indicative of cardiac tamponade. This condition results from the accumulation of fluid in the pericardial space, leading to increased pressure around the heart and hence, reduced cardiac output. Further, the patient's nonspecific ST-T changes on ECG could be associated with pericardial disease, including pericarditis and cardiac tamponade. His presenting symptoms of exercise intolerance, dyspnea and coughing are in line with the reduced cardiac output that typically results from severe forms of cardiac tamponade. Interestingly, despite the resolution of apical and perihilar infiltrates and a normal-sized heart on his chest X-ray, these features should not be used to rule out the likelihood of cardiac tamponade given their limitations to depict pericardial fluid accumulation especially when it’s a slow process like in tuberculous pericarditis. (4) CONCLUDING: Given the high index of suspicion for cardiac tamponade as the etiology of this patient's deteriorating clinical condition, it's reasonable to conclude that the most appropriate next step in the evaluation of this patient should revolve around confirming this diagnosis. Therefore, (E) Ordering echocardiography becomes the most likely and preferred choice as echocardiography is the most useful diagnostic tool for circumstantially investigating such a condition of suspected pericardial tamponade. (5) RESPONSE: I recommend pursuing option E - performing an echocardiography. This will not only help confirm our suspicion of cardiac tamponade but may also show the approximate amount of pericardial effusion. Further treatment, typically pericardiocentesis, can then be guided based off of the results. Preceding with this is crucial because this condition can lead to a critical decrease in cardiac output contributing to shock, and therefore, immediate diagnosis and management is paramount.

**Facilitator** The consensus among the AI responses is that the most appropriate next step in managing the patient's condition is to order an echocardiography (Option E). The patient's symptoms, including shortness of breath, cough, right upper quadrant pain, and lower extremity edema, despite ongoing treatment for tuberculous pericarditis, suggest a possible complication such as constrictive pericarditis. This condition, characterized by the thickening and calcification of the pericardium, restricts the heart's ability to fill and expand, leading to systemic congestion. The patient's clinical presentation, including jugular venous distention, tender liver, and lower extremity edema, further supports this suspicion. An echocardiography is the most definitive method to diagnose constrictive pericarditis and differentiate it from other potential causes. Therefore, it is the preferred choice for further investigation of the patient's condition.
